# Evolution of Model Compounds and Functional Group Compositions for Molecular Dynamics Simulations of Aged Asphalt Binder

**DOI:** 10.3390/molecules30224476

**Published:** 2025-11-20

**Authors:** Edgar A. O’Rear, Liangliang Huang, Musharraf Zaman

**Affiliations:** 1 School of Sustainable Chemical, Biological & Materials Engineering, University of Oklahoma, Norman, OK 73019, USA; hll@ou.edu; 2 School of Civil Engineering and Environmental Science, University of Oklahoma, Norman, OK 73019, USA; zaman@ou.edu

**Keywords:** asphalt binder, molecular dynamics, functional groups, oxidation, sustainability, molecular basis set

## Abstract

To apply molecular dynamics (MD) simulations in the study of virgin asphalt binder, researchers have relied on basis sets of representative model structures from the SARA categories of saturated aliphatics (S), naphthenic aromatics (A), polar aromatics or resins (R), and asphaltenes (A). The evolution of these model compounds for MD of binder is reviewed with emphasis on addition of oxidized species for simulations of recycled aged binders. The level and type of oxygen functional groups in many MD simulations are not consistent with reported findings. Oxidation of primary, secondary, and tertiary benzylic carbons has been used as a rational approach to generate an extended basis set with functional groups reflecting ageing of virgin binder model compounds. Moieties known to be present in aged binder, though not wholly represented in prior work, include carboxylic acids, ketones, alcohols, anhydrides, and sulfoxides. A specific modified basis set for oxidized asphalt binder is proposed along with a methodology for generating other oxygen-consistent basis sets from virgin binder structures. An example illustrates how selection of compounds from the modified basis set and their amounts can be used to match observed functional group compositions. The objective of this approach is more realistic representation of the molecular interactions between aged asphalt binder structures and those in a waste cooking/motor oil, for example, used to rejuvenate the rheological properties of a binder.

## 1. Introduction

In terms of potential mass salvaged, the asphalt concrete in paved roads in the United States and around the world represents one of the greatest opportunities for sustainability. On over 90% of the roads in this country, asphalt pavement exists primarily as rock or “aggregate” held together by an asphalt binder, which is a petroleum product in most cases. To recycle old asphalt pavement, it is necessary to overcome the deterioration of its properties with ageing and distresses. Asphalt concrete degrades with time due to weather and traffic loads, and more fundamentally to oxidation and loss of volatile components [1]. The stiffer physical properties lead to cracking and fragmentation with eventual failure of the pavement, including premature failure. Poor properties after ageing have been cited as a major impediment to recycling of binder [2]. Rejuvenators can restore rheological properties of asphalt binder so that recycling becomes economically and functionally attractive.

Reuse of asphalt binder necessitates chemical additives, at the very least a rejuvenating agent. Also of interest is incorporation of other materials and additives. Reclaimed asphalt pavement (RAP) represents a sustainable resource [3]. In addition to reuse of the binder itself from RAP, it has been proposed that a variety of other waste materials might be applied beneficially to paving roads and highways rather than taking up space in a landfill. Some of the wastes considered include ground tire rubber, recycled asphalt shingles, slag, coffee grounds, polymers, engine oils, waste toner, and waste cooking oils (WCO). Of these, the oils and waste toner stand out because they have been shown to function effectively as rejuvenators [4,5,6,7] which benefit rheological behavior. Underlying the performance of rejuvenators and other materials in a pavement will depend on favorable molecular interactions with recycled oxidized asphalt binder. How to employ fundamental chemistry for their successful integration into aged asphalt is a formidable challenge. This means, at the molecular scale, considering the specific functional groups present that influence macroscopic properties. The chemical transformation of virgin binder with ageing requires accommodating the presence of oxidation products like carboxylic acids, ketones, and alcohols and the greater role of polar species in analysis using molecular dynamics. With inclusion of remnant virgin binder structures for more realistic functional group concentrations, the complexity of molecular interactions of binder with any added material increases significantly.

Molecular dynamics simulations hold promise as a tool to obtain molecular-level understanding of interactions between asphalt binder and additives. This requires creating a robust system of model compounds to mimic aged asphalt binders in silico. We propose advancing the field with a new basis set of model compounds for MD simulations of aged asphalt binders. The set contains often overlooked functional groups that are known to be generally present due to oxidation. Structures in the basis set have been derived by considering oxidation chemistry of organic compounds with structures proposed by others for virgin asphalt binders as a starting point. Use of a basis set allows selection of component molecules to accommodate varying degrees of oxidation and composition deriving from the original virgin asphalt cement. A methodology is suggested so that researchers can develop their own basis set for aged binder if desired.

The objective of this study is to advance the structures and compositions used in simulations of aged asphalt binder. Prior work on aged, oxidized asphalt binder has not accurately represented the range of known functional groups and their concentrations [8]. This is important because, in addition to the usual expectation of structure–property relationships, dependence of physical properties on functional group composition for asphalt follows from viscosity relationships reported by Peterson [9,10,11], a leading researcher for many years on the chemistry of asphalt. Moreover, using molecular structures closer to their actual atomic structure is conceptually appealing and in line with recent trends. Lastly, in this study, we demonstrate how a researcher using MD can mix and match elements from the basis set to align functional group and SARA compositions with real aged asphalt binder samples.

### 1.1. Asphalt and MD Simulations with Basis Sets of Representative Structures

Successful prediction of physical properties for aged asphalt binders can be challenging as asphalt is a very complex substance. Its composition includes millions of different molecules which vary greatly from one sample to the next depending on the source of the petroleum and its processing at the refinery. It has been estimated that asphalt has 10^5^–10^6^ different molecules [12]. Thus, the explicit representation of exact chemical species present in any asphalt binder is not feasible. Nonetheless molecular dynamics has become an active area of research on asphalt and a valuable tool with many papers appearing in recent years [13].

Simulations became possible as chemists suggested specific examples of chemical compounds in asphalt cements. Researchers then created representative structures to enable application of MD to the study of asphalt binder. Early work focused on asphaltenes and their aggregates since they primarily determine viscosity and viscoelastic properties. Pacheco-Sánchez et al. used four structures representative of asphaltenes to investigate their orientations within aggregates [14]. Later work on molecular orientation by Zhang and Greenfield [15] placed asphaltenes in a more realistic environment with compounds selected for each of saturate, aromatic, and asphaltene fractions. Specifically, their compounds were n-docosane (C22), 1,7-dimethynaphthalene, and either of two asphaltenes proposed by Artok et al. [16] or by Groezin and Mullins [17]. Building on Mullins’ asphaltene molecules, Zhang and Greenfield [15] and Li and Greenfield [18] progressed from a C22-alkane and 1,7-dimethylnaphthalene model mixture with two asphaltenes to one containing a total of 12 representative molecules. Structures proposed for molecular dynamics simulations have been reviewed by Qu et al. [19].

These component systems with actual chemical structures have served as a platform to launch molecular dynamics studies on properties of asphalt. One can vary composition with selected combinations of molecules from this basis set to simulate asphalts from different sources. Wang later expanded the set with additional aromatic, resin, and asphaltene molecules [20]. These systems provide a consistent framework for investigating properties and performance of virgin asphalt in different situations.

**Figure 1:**
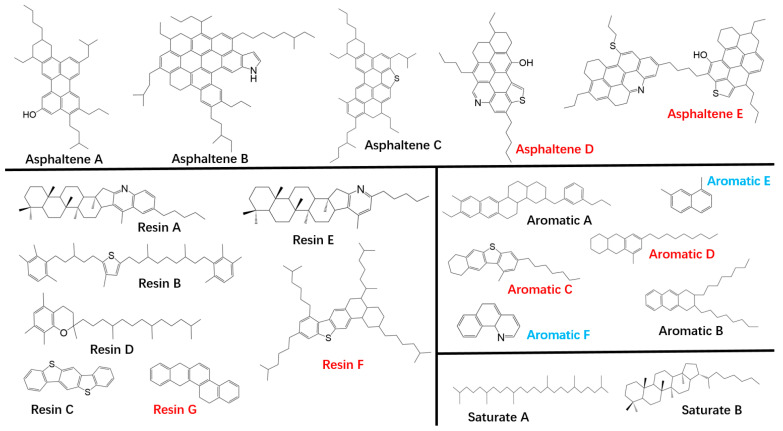
A combined basis set comprised of virgin asphalt binder model compounds developed by Zhang and Greenfield (blue), Li and Greenfield (black) and Wang et al. (red) [22].

One approach has been to capture asphalt chemistry by defining broad categories of molecules. This method relies on generic SARA fractions or other asphalt separations [21]. These groups with their different polarities have been related to the colloidal nature of asphalt. For example, resins act as dispersing (peptizing) agents for large asphaltene aggregates within the more nonpolar maltenes fractions. While there has been some success in relating properties to the SARA composition, the number of components and their imprecise definition limits its utility for conceptual advances on the basis of fundamental chemical principles. In seminal papers, Zhang and Greenfield [15] and Li and Greenfield [18] made a significant step forward when they proposed sets of model compounds—eventually 12 actual molecular structures—representing the SARA fractions. These molecules with structures appropriate to the various fractions have been used by a number of researchers for molecular dynamics studies of asphalt. Wang [20] modified the composition of the Li and Greenfield model, principally with the addition of aromatic and resin molecules to accommodate high sulfur asphalts. The combined molecular structures from these groups (Figure 1) enabled the study of physical properties of virgin asphalt binders with molecular dynamics [22].

For investigation of unoxidized asphalt binder properties by MD, binder composition can be varied by selecting the numbers of molecules from each SARA fraction. One such property, self-healing, is of great importance as cracks evolving from microcracks can lead to failure of pavements. Using Zhang and Greenfield’s three-component model structures and others proposed by Jennings [23], Bhasin et al. employed molecular dynamics to investigate diffusion across a microcrack and better understand self-healing in asphalt [24]. That included, for example, the effect of SARA composition on the self-healing property. Taking an unusual approach in the study of micro-healing, Sun et al. carried out MD simulations by constructing a single “average molecule” for asphalt and thereby implicitly treating asphalt as a pure compound [25]. A single structure would seem likely to fail in capturing the entropic complexity of a multicomponent system and thus unable to capitalize well on the ability of molecular dynamics to investigate the effects of temperature. Notwithstanding, the researchers concluded that the optimal temperature range for self-healing was 40.3–48.7 °C. Molecular dynamics has also been used to investigate the formation of nano-aggregates of asphaltenes. Samieadel et al. found that nano-aggregates of a polycyclic aromatic asphaltene were reduced with the addition of undecane wax, which provided possible insight into the underlying mechanism about how commercial wax products function as a warm mix asphalt additive [26]. MD has also been used to examine a number of other properties including glass transition temperature, viscosity, bulk modulus, and adhesion [13,27,28]. These examples illustrate the potential of MD in real world applications.

### 1.2. Molecular Dynamics of Aged Asphalt and the Importance of Functional Groups

The sophistication of these simulations has improved as better representations of virgin asphalt with model compounds appeared. Most reports emphasize oxidation as responsible for changes in physical properties with ageing, though some consider change in SARA composition with volatilization. Ding et al. employed Zhang and Greenfield’s three-component model to study ageing and the diffusion of rejuvenator in RAP and virgin binders [29]. To account for ageing, they reduced the number of C22 maltene molecules in the simulation and increased the number of asphaltenes. This failed to account, however, for the key factor of oxidation by modification of structures with oxygen atoms.

An early molecular dynamics study with oxidation was carried out by Tarefder et al. using a system comprised of three structures taken from the literature, the asphaltene of Groezin and Mullins [17] and two resin species based on Venezuelan crude oil [30]. Saturates were not included, apparently based on recognition that most of the oxidation is known to occur in the polar resins and asphaltenes. They investigated the glass transition temperature T_g_ with oxidation. Above T_g_, asphalt is more deformable, which is of interest because some have suggested that it is tied to low temperature cracking. T_g_ can be found as a discontinuity in the rate of change of specific volume with temperature. Tarefder increased the number of oxygen atoms in the asphaltenes and resins from 0.1% to 1%, 12%, 23%, and 46.5%. They found that glass transition temperature was not affected by oxygen levels below 20% and decreased with higher percentages. A shortcoming of this work was the absence of a description of oxidation states and functional groups present. Oxygen character differs from one functional group to the other. Interaction between molecules having more polar hydroxyl or acid groups will be substantially different from those with ketones.

Other researchers advanced the model for aged asphalt by “oxidizing” the Li-Greenfield set of 12 molecules including one recent report where oxidation sites were guided by quantum chemistry calculations [31]. In most of these new models, investigators created sulfoxides and ketones, thereby indicating an appreciation of the known relationship of viscosity to these two functional groups [9,10]. Placement of ketones in the design of oxidation products was guided to a degree by chemistry. In particular, Dorrence et al. had noted the susceptibility of benzylic hydrogens to oxidation to form ketones [32] so that all secondary benzylic carbons for all structures were converted to a ketone moiety. This fit nicely with the known relative reactivity of resins and asphaltenes being much greater than that of the naphthenic aromatic and saturate fractions [37:42:7:1]. This produced a set of base molecules which were mixed in different combinations by several researchers to simulate different aged asphalt samples according to SARA composition. With this approach, Pan and Tarefder showed oxidation resulted in greater strength of intermolecular bonds with increase in density, viscosity, and bulk modulus [33]. Qu and colleagues carried out MD with the same base set to propose correlations between micro- and macroscopic properties [34]. To simplify the asphalt model for a study on oxidation, Cui et al. employed four average molecular structures in their simulations, one for each SARA fraction [35]. Their model was used to investigate formation of ketones and sulfoxides. G. Xu et al., in looking at the effect of ageing on physical properties [36], found that oxidation created a higher activation energy for self-healing. Xu et al. also carried out molecular dynamics simulations of their own selection of model compounds to investigate diffusion of rejuvenator into binder with RAP species [37]. Wang et al. examined, with and without waste cooking oil, cohesive energy density and surface free energy of aged asphalt binder which reflect its strength and crack resistance [5]. These studies help to demonstrate the potential of molecular dynamics for study of aged asphalt.

**Figure 2:**
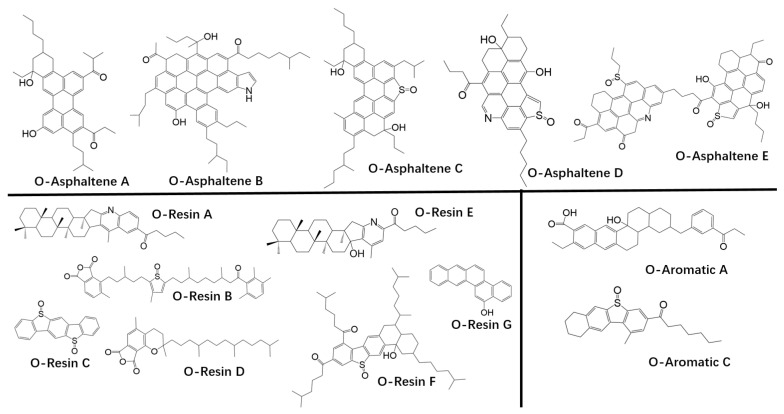
Proposed oxidized forms of the virgin asphalt compounds designated with prefix O. Oxidation creates ketones, sulfoxides, alcohols, enols with aromatization (ketone tautomers), anhydrides, and carboxylic acids [22].

There are, however, some issues with the aforementioned oxidation models which led us to propose expanding the basis set with oxidized structures (Figure 2). With only ketones and sulfoxides present or none specified at all, the absence of other oxygen-containing functional groups is a limitation. Other functional groups do form with oxidation and some are important. Alcohols, for example, have been shown to have a similar effect on viscosity as ketones [11], but no alcohols are present in any of these models. We also note that the ketone levels with the structures employed are too high by an order of magnitude. Using the molecular composition (i.e., the number of each basis set molecule in the simulation) in these papers and assuming an approximate asphalt density of 1.00 kg/L, we calculated the ketone content of each to be on the order of 5 mol/L. This does not compare well to experimental values of 0.50, 0.55, 0.58, and 0.77 mol/L found by Petersen for four different field aged asphalt samples [38]. The calculated sulfoxide content ranged from 0.34 to 1.05 mol/L compared to 0.30, 0.30, 0.29, and 0.18 mol/L.

**Figure 3:**
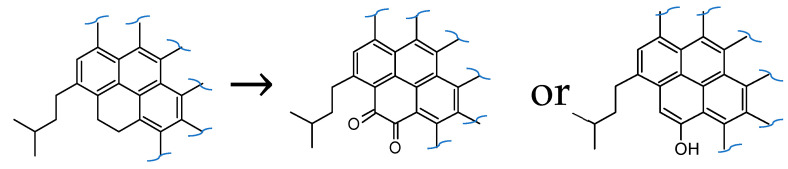
Questionable diketone product of Asphaltene B in oxidized secondary benzylic carbon model.

Moreover, we question one of the structures resulting from simply converting all secondary benzylic carbons to the ketone without consideration of organic chemistry. The diketone structure of the oxidized asphaltene-pyrrole (Figure 3) is questionable since oxidation to the first ketone may cause conversion to the phenol tautomer with formation of a pyrene core structure.

**Scheme 1:**
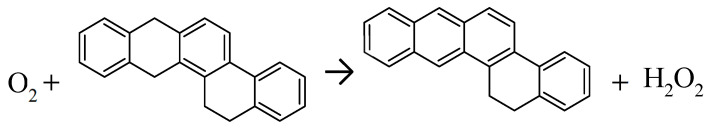
Aromatization as a fast component of asphalt binder oxidation.

**Scheme 2:**
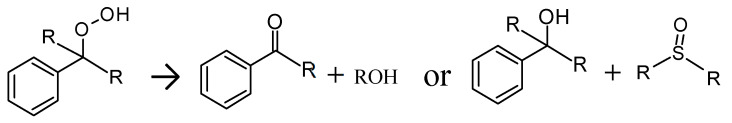
Oxidation of a tertiary benzylic hydrogen with a hydroperoxide intermediate.

Recently, M. Xu et al. developed a new oxidized asphalt model with significant advances based on chemistry [39]. As opposed to having only oxidized molecules, some of the original virgin asphalt structures were also included in aged asphalt samples. The combination of unoxidized and oxidized species creates molecular interactions similar to the presence of partially oxidized structures while keeping the total number of species manageable. Xu used an expanded basis set to include structures proposed by Wang et al. [20]. Of particular note is a more sophisticated effort to consider oxidation chemistry in forming structures found in aged asphalt. Petersen’s theory of fast and slow oxidation reactions [40] was employed to create reaction products. The fast reactions involve aromatization of polycyclic structures like resin G (Scheme 1) with byproduct hydrogen peroxide or other peroxide intermediates acting to oxidize thioethers to sulfoxides. Xu is the first to our knowledge to consider an aromatization component. Formation of polycyclic aromatics contributes to increased viscosity by greater association with the asphaltene fraction via π-bonding [11,41]. Peterson’s reaction scheme for oxidation of asphalt involved a slow reaction phase proceeding through formation of the hydroperoxide of a tertiary benzylic species which can decompose to an aryl ketone and an alcohol or a benzylic alcohol and other oxidation products such as a sulfoxide (Scheme 2). Considering all possible products of each tertiary benzylic carbon in the basis set, Xu et al. created an additional 21 possible product molecules [39]. Thus, their model for oxidized asphalt now included alcohols as well as ketones. Overall, up to 45 different species existed for the aged sample. Three types of asphalt used widely in north China were matched in SARA and H, N, O, and S compositions. Simulations were carried out to model AFM adhesion force and nano-hardness experiments.

M. Xu’s work [39] made valuable contributions to the field and yet we see opportunities on which to base improvements. As in all prior research, his study failed to consider measured values of functional group concentrations in aged asphalt. This is important because concentration of polar moieties will help determine the number of strong molecular interactions. We carried out calculations to estimate the functional group composition of Xu’s samples. Ketone concentrations were 0.13, 0.56, and 0.12 mol/L and sulfoxide levels were 0.43, 0.18, and 0.69 mol/L. While these values are an improvement, the agreement is not particularly good with Petersen’s findings. Moreover, we take issue with the choice of oxidation products as will be explained in the next section.

## 2. Construction of an Oxidized Basis Set

### 2.1. An Aged Asphalt Basis Set with Functional Groups Directed by Oxidation Chemistry

As previously noted, much of what is known about the oxidation of asphalt comes from the experimental and theoretical work of J. Claine Petersen at Western Research Institute. Petersen maintained that physical properties depended on “chemical functional types” [42]. A focus on functional groups can capitalize on much of his research while adding a level of structural representation beyond solely consideration of elemental analysis and SARA fractions. It provides greater access to relating structures in molecular dynamics simulations to experimental characterizations of asphalt binders.

**Figure 4:**
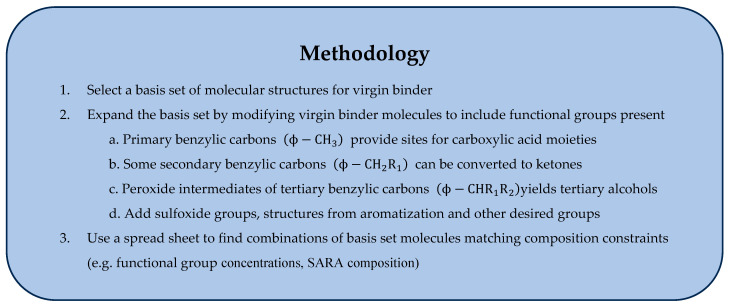
Procedure for construction of a basis set for MD simulations of aged asphalt binder of known functional group composition. Symbol ϕ represents an aromatic ring structure.

A starting point for the development of oxidized asphalt molecules in a basis set is virgin asphalt binder structures proposed by Zhang and Greenfield, Li and Greenfield, and Wang et al. We have modified their basis set molecules to yield products with functional groups expected after ageing (Figure 2). At least ten different functional groups have been identified in asphalt and aged asphalt [11,38]. These moieties include polynuclear aromatics, ketones, sulfoxides, alcohols, anhydrides, and carboxylic acids, all of which form with ageing while the first and last of these also occur naturally. To incorporate them into the basis set, we searched the virgin asphalt structures for oxidation sites appropriate to the various functional groups. More of these sites exist in the resin and asphaltene fractions in line with the fact that they have much higher susceptibility to oxidation. For example, tertiary benzylic sites (Figure 1: Aromatic A; Resins E, F; and Asphaltenes A–E) are very reactive so these were converted to the alcohol. Diverging from Xu’s approach, we eliminated the ketone products resulting from the hydroperoxide degradation of Scheme 2 after reviewing the literature (See item 2 below). To account for the presence of ketones, we converted secondary benzylic carbon sites (Aromatic A and C; Resins A, B, E, and F; Asphaltenes A, B, D, and E), though not all such sites were modified. An aryl methyl group or primary benzylic carbon on Aromatic A was envisioned as being oxidized to the aldehyde which is very susceptible to further oxidation yielding a carboxylic acid functional group. Adjacent aryl methyl structure on Resins B and D created sites for the anhydride moiety also reported for aged asphalt. We note that the oxidation of primary, secondary, and tertiary benzylic carbons is consistent with the full range of functional groups in the literature. Thioethers and thiophenes (Aromatic C; Resins B, C and F; and Asphaltenes C, D, and F) have been oxidized to the sulfoxide. These 14 oxidized molecules (Figure 2) with the 20 virgin asphalt structures constitute an expanded basis set for aged asphalt. The set is clearly not unique with variations of the approach possible, but with samples constrained by functional group concentration, the proposed structures should yield a good approximation to the spectrum of intermolecular forces in a molecular dynamics simulations. Figure 4 summarizes the steps in the methodology.

### 2.2. Principles Used to Construct an Expanded Basis Set from Virgin Asphalt Structures

i. A broad range of known chemical moieties in oxidized asphalt should be included. At least 11 different functional groups have been identified in aged asphalt including six species formed by oxidation. Included are ketones, sulfoxides, anhydrides, carboxylic acids, alcohols, and polynuclear aromatics [38], some of which have been omitted in prior models.
ii. Tertiary benzylic carbons will form on oxidation benzylic alcohols only. Petersen’s scheme for the slow phase degradation of the tertiary benzylic hydroperoxide yields both alcohol and ketone products [40]. While this is correct, the literature indicates the ketone is a minor product. The thermal free radical decomposition of the tertiary benzylic hydroperoxide formed from cumene yields over 80% of the alcohol product [43]. As such, we believe M. Xu et al. over represented this type of ketone component [39]. We eliminate a corresponding 14 of the 45 structures by focusing on the alcohol product.
iii. Oxidation of secondary benzylic carbons yields ketones. Petersen’s dual (fast and slow) oxidation mechanism provides for the formation of ketones only from degradation of tertiary benzylic carbons. If this were true, one would expect to see the tertiary benzylic alcohols being formed contemporaneously with the ketone through their mutual hydroperoxide intermediate. Kinetic data [11] clearly shows that this is not the case. Ketone formation continues much longer after alcohol attains a constant value. This means ketones are being formed by other reactions. M. Xu only forms ketones through the tertiary benzylic hydroperoxide. Strong evidence indicates oxidation yielding ketones at the benzylic position [32] which in combination with the kinetic data for alcohols points to secondary benzylic carbons. Thus, we create ketone moieties in basis set structures from the secondary benzylic carbon molecules of the virgin asphalt models. Many, but not all such sites were oxidized.

We do face a challenge in resolving one piece of available knowledge which likely influenced Petersen’s and Xu’s thinking. There is a report based on proton NMR that tertiary benzylic carbons constitute the major benzylic structure in asphalt [44]. The ability for NMR to discern differences in benzylic protons with a high degree of confidence has to be suspect when looking at a complex mixture like asphalt with a wide range of chemical shifts for benzylic protons and with splitting due to coupling likely for many of the proton signals. We note that many other groups have seemingly discounted this finding in posing structures for asphalt.

iv. Primary benzylic carbons in the virgin asphalt structures offer sites susceptible to oxidation for carboxylic acid and anhydride formation. Similarly, a methyl substituent on an aromatic ring with benzylic hydrogens can be oxidized to the aldehyde and then further to a carboxylic acid group. Aldehydes readily convert to the corresponding carboxylic acid in air. A set of adjacent aryl methyl moieties in Resin B and D of the virgin asphalt model provided a logical site to insert anhydride functional groups.
v. Thioethers and thiophene moieties are converted to sulfoxide species. Experimental work shows that sulfoxides act to help determine the viscosity of aged asphalt. Except for some of the earliest models, they have often been included for molecular dynamics simulations.
vi. A multiring molecular component allows for oxidation to a polynuclear aromatic structure. Some multiring molecules with both aromatic and aliphatic portions (e.g., Resin G, Figure 1) can be oxidized to a polynuclear aromatic species. Their formation followed by association with asphaltenes and polar aromatics through π-π bonding is thought to play a role in increasing stiffness.

## 3. Results and Discussion

This study produced a basis set of molecular structures comprised of species from the literature for virgin asphalt binder as shown in Figure 1 supplemented with species derived by their oxidation (Figure 2) to include ketones, carboxylic acids, alcohols, sulfoxides, and other functionalities. We believe that including consideration of functional groups and setting their concentrations at realistic levels will advance the MD simulations of both virgin and aged asphalt binder. Considering organic chemical reactions, we prepared a modified basis set for aged asphalt which might be called a functional group model (FGM). Drawing on the 34 species in this basis set (Figure 1 and Figure 2), one can create mixtures to satisfy SARA or functional group constraints of oxidized asphalt binder. The basis set is not unique and variations on structures in the oxidized components of the basis set might prove to be superior using the same approach. For modified asphalts, oxidation of additives may be necessary. An additive like styrene-butadiene-styrene copolymer (SBS) used to promote adhesion might be included in a basis set. For example, Xie et al. considered the effects of SBS oxidation and included oxidation products in a MD simulation as part of a study on adhesion of SBS-modified asphalt to aggregate [45].

Development of oxidized species for aged binder was largely influenced by Dorrence et al. with formation of ketones at benzylic carbons. Often, all such sites were converted yielding an arbitrary concentration of the ketone functional group. Other known functional groups in aged binder were not included. Recent articles on molecular dynamics simulations of aged asphalt show that such problems persist. Moreover, representations of the aged asphalt binder tend to employ only structures that have been oxidized, with no virgin binder compounds present except for saturates [45,46,47,48,49]. An exception is work by Cui et al. who utilized several different basis sets for simulations to reflect the degree of oxidation [50]. In all these studies, the concentration of the oxygen functional groups, ketones, and sulfoxides, lacks justification. Based on Petersen’s work and our calculations, the level of ketones is too high, meaning addition of residual virgin binder species should be considered. There is an opportunity to advance the field with greater consideration of the effects of oxidation.

Effective MD simulations depend, in principle, on adequate representation of the variety of molecular interactions between species structural components and the extent of those interactions. Molecular architectures including atoms and the functional groups present in the system will determine the types of interplay between species while composition will influence the likelihood of each. For more typical simulations of a system with few components where the structure of molecules and their numbers can be specified, these factors can be addressed directly in silico. In the case of a complex mixture like asphalt, substituted model compounds ideally replicate the types and frequency of interactions taking place so that physical behavior of the system will correspond to experimental observations.

The key feature of this work is providing an improved means of capturing the types of molecular interactions that should be present between the different atoms and functional groups. Furthermore, choosing the numbers of each compound in the basis set can affect the likelihood of a particular interaction in a simulation. It is important to realize that experimental analyses of chemical group composition in aged asphalt provide data relevant to the type and number of such interactions. Thus, experimental values for the concentration of different moieties can guide selection of the component compounds from the basis set for a particular binder.

To illustrate how FGM can be used to develop a composition fitting experimental functional group levels, we have constructed a combination of basis molecules satisfying constraints from the literature. Levels of four functional groups from four aged asphalts collected from widely different sources have been reported [38]. Nominal concentrations found were 0.60 mol/L ketone, 0.30 mol/L sulfoxide, 0.025 mol/L anhydride, and 0.007 mol/L carboxylic acid. Data on the level of alcohol moiety was not provided, but based on details in the dual oxidation mechanism, we expect it to be on the same order as the sulfoxide.

**Figure 5:**
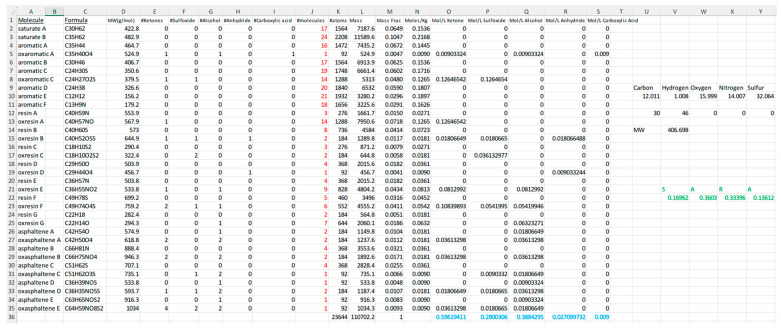
Spreadsheet tool for generating a mixture of model compounds for molecular dynamics simulations of virgin or aged asphalt binder. Molecular designations indicate the category (saturates, aromatics, resins, asphaltenes, or their oxidized counterparts) with a letter naming a specific compound from Figure 1 and Figure 2. A representative composition is shown with the number of each species indicated in red (i.e., 17 molecules saturate A; 24, saturate B; 16 aromatic A, etc.). Functional group and SARA compositions for this binder model are calculated and shown (blue and green, respectively).

The composition shown in Table 1 is not unique. Other combinations of this aged asphalt basis set or others could meet composition constraints. A spreadsheet tool (Figure 5) was developed to aid selection of compounds and find a composition matching the reported functional group concentrations. Table 1 presents a sample composition with the number of each basis set compound shown. On entering the number of each molecule in the spreadsheet (shown in red), one obtains a SARA composition of 17% saturates, 36% aromatics, 33% resins, and 14% asphaltenes (green). In addition, sample A1 yields concentrations of 0.5962, 0.2800, 0.3884, 0.0271, and 0.0090 mol/L for ketone, sulfoxide, alcohol, anhydride, and carboxylic acid, respectively (blue). Figure 5 shows the spreadsheet tool with data on a line for each of the 34 basis structures (molecular formula, molecular weight, #ketones, #sulfoxides, #alcohols, #anhydrides, and #carboxylic acids). Chang et al. employed this aged binder composition to investigate waste cooking oil as a rejuvenator using molecular dynamics [22]. Other virtual samples for MD simulations of aged (or virgin) asphalt can be prepared by selecting other combinations of the FGM set.

Functional group concentrations in the spreadsheet were calculated as shown in the following. First, total atomic mass was calculated for the specified composition:

(1) $${Mass}_{TotalAtomic}=\sum N_{i}*{MW}_{i}$$

where N_i_ is the number of species i indicated in red times its molecular weight MW_i_. The mass fraction of each component w_i_ was obtained using the total atomic mass:

(2) $$w_{i}=\frac{N_{i}*{MW}_{i}}{{Mass}_{TotalAtomic}}$$

From which the number of moles of a basis compound in mass M = 1000 g (or 1 L) was determined. An overall density of 1000 g/L was assumed:

(3) $$N_{Moles,i}=\frac{w_{i}*M}{{MW}_{i}}$$

The total number of moles of a functional group (e.g., ketone) in 1 L was found from:

(4) $$N_{TotalKetone}=\sum N_{Ketone,i}*N_{Moles,i}$$

The result is indicated in blue in Figure 5 as concentration for this model binder composition.

The literature on molecular dynamics shows the value of examining a broader range of functional groups that may be important, especially at interfaces. Zhu et al. found significant differences between studying oxygen functional groups in the hydration layers between coal and quartz with molecular dynamics [51]. Weakly polar species like ethers (-OCH_3_) and aldehydes (-CHO) result in more diffuse hydration compared to polar carboxylic acids (-COOH) and alcohols (-OH). Others have examined oxygen functionalities on modified surfaces of graphene oxides which are of interest in nano-composites. Dou et al. assessed the wettability of graphene oxide functionalized with hydroxyl or epoxide moieties at different surface coverages [52] while Hou et al. examined the adsorption of calcium and sulfate ions when graphene oxide was modified with carboxylate (-COO^−^), carboxylic acid (-COOH) or hydroxyl moieties (-OH) [53]. Results of an investigation of binder adhesion to aggregate showed the significance of oxygen moieties in the water-stripping of asphalt pavement [49]. The oxygen-containing functional groups (ketones and sulfoxides) participated in hydrogen bonding with water to weaken adhesion. Unfortunately, alcohol was not considered. Including alcohols among the functional groups in the simulation would have represented both proton donor and acceptor contributions to hydrogen bonding by the binder.

Beyond improving model compounds and aged-binder compositions, two additional constraints shape what MD can and cannot tell us: how well the force-field captures interatomic interactions, and practical system-size/time-scale limitations inherent to simulating a highly polydisperse, multi-component petroleum mixture. Oxygen-bearing functional groups differ in bonding environments, polarity, and electronic structure; these differences map directly to parameterization choices in an atomistic force-field and can influence density, cohesive-energy density, diffusion, and key RDFs. Continued progress in data-driven force-field development [54] should improve fidelity without changing the workflow proposed here.

Application of MD to asphalt remains challenging precisely because the “true” molecules are not known and are too numerous to model explicitly; representative compounds, therefore, substitute for actual species. This makes asphalt a genuinely multidisciplinary problem for physical/analytical/organic/theoretical chemistry and underscores an ongoing need for better compositional constraints for aged binders. We note encouraging work using carbonyl index as a practical spectroscopic gauge and emphasize the value of anchoring MD inputs to quantitative functional-group concentrations wherever possible [55].

Three specific constraints and how to mitigate them are: *Force-field dependence.* Computed density, CED, diffusion, and RDFs for polar oxygenated species are sensitive to the choice of all-atom parameters and charge models. To reflect this uncertainty, we recommend recomputing core observables under at least two independent all-atom parameterizations and reporting ranges not single values. The proposed FGM is force-field agnostic, so future improvements, such as machine-learning force-fields [54], can be adopted without modifying the FGM workflow. *Avoiding overfitting of functional-group concentrations.*

To avoid tuning a mixture to one target and losing generality, composition selection in the spreadsheet should be treated as a multi-objective match with tolerance bands, simultaneously constraining: (a) functional-group concentrations from the literature, (b) SARA fraction ranges, (c) elemental composition (H/N/O/S), and (d) bulk density. A mild regularization can discourage large departures from the virgin-binder composition. We further suggest cross-validation against at least one property not used in fitting (e.g., a plausible carbonyl-index trend) to make sure mixtures are not over-tuned to the functional-group targets alone. *Non-uniqueness*. Multiple mixtures can satisfy the same constraints. We, therefore, recommend generating an ensemble of feasible compositions and presenting key MD outputs as mean ± variability across that ensemble. For transparency and reproducibility, at least two distinct mixtures that meet the same constraints should be reported, together with the spreadsheet used to generate them. This implements and operationalizes our statement that the basis set is not unique.

The limitations of existing models related to FGM can be summarized as not taking advantage of certain known chemistry to form more realistic basis sets. Literature on asphalt binders includes available information for greater consistency in structure with the actual molecules present. In a review on molecular dynamics, Wang et al., like Petersen [38], present valence bond images of typical structures in asphalt binders [21]. These molecular structures, though mislabeled, confirm the functional groups described by Petersen and others. The articles by Wang and Petersen serve as resources to help overcome this limitation. Models also fall short generally in using arbitrary levels of oxidation that are not based on experimental data. The problem arises in part from the limited availability of measurements related to functional group concentrations with ageing. Studies combining MD simulations with experimental measurements can help address this shortcoming.

We emphasize that the FGM model is compatible with other approaches. For example, SARA composition in MD simulations can be adjusted by the number of each species in the fractions, independent of the choice of functional groups in those species as guided by FGM. We recommend using a spreadsheet tool with the basis set of choice to help address composition for simulations. The value of using experimental SARA analysis such as that by Sultana et al. and others on the relationship of physical properties to SARA composition [35,56,57] should not be lost and might be better simulated with data on functional groups. Incorporating the diversity of chemical moieties at validated levels offers the prospect of improving MD simulations.

Further variation and improvements in basis sets used for aged asphalt binder can be anticipated. Those sets may include use of FGM structures in development. If they do, the mechanism of continued evolution of model molecular structures will involve assessing the success of their implementation in MD simulations.

Future work should include quantitative measurements of the concentrations of functional groups, especially for aged binder. There are opportunities and needs for more research on the structural chemistry of binders to strengthen the connection of model compounds to the species present in a real sample. Continued advancement in force-field representation is essential to achieve gains possible with more realistic compositions in aged binders.

## 4. Conclusions

Knowledge about the chemical compounds and composition of binders has been essential to the evolution of surrogate structures employed in MD simulations. Virgin binder model species reflect the SARA composition while aged binder is less well-characterized overall. Past and current studies have failed to recognize the many different functional groups found in analyses of aged binders by Petersen. In this work, commonly used basis set molecules for virgin asphalt binder have been modified at benzylic carbons to incorporate carboxylic acids, alcohols and other oxygen-containing functionalities.

A proposed basis set includes virgin binder species in order to obtain realistic levels of functional groups in aged binder. Calculations indicate that concentrations of ketones in many MD studies of aged binder are too high and not consistent with experimental findings by Petersen. A spreadsheet tool can be used to examine different combinations of basis set elements which are not unique for a target concentration. The field would benefit from additional research on the composition of aged asphalt binder.

## Figures and Tables

**Table 1:** Sample binder meeting a specified composition * (number of molecules).

Saturates	Aromatics	O-Aromatics	Resins	O-Resins	Asphaltenes	O-Asphaltenes
A 17	A 16	A 1	A 3	A 14	A 2	A 2
B 24	B 17		B 8	B 2	B 4	B 2
	C 19	C 14	C 3	C 2	C 4	C 1
	D 20		D 4	D 1	D 1	D 2
	E 21		E 4	E 9	E 1	E 1
	F 18		F 5	F 6		
			G 2	G 7		

* Letter designates a basis set molecule within a category group. Refer to Figure 1 for the molecular structure corresponding to (say) Aromatics C and Figure 2 for its oxidized form under O-Aromatics C. Each species has the number of molecules in this binder model indicated (e.g., 19 molecules of the Aromatics C species and 14 molecules of O-Aromatics C.

## Data Availability

No new data were created or analyzed in this study. Data sharing is not applicable to this article.
